# Densely Distributed Multiple Resonance Modes in a Fan-Shaped Plasmonic Nanostructure Demonstrated by FEM Simulations

**DOI:** 10.3390/nano9070975

**Published:** 2019-07-04

**Authors:** Qiong Wang, Zhengbiao Ouyang, Qiang Liu, Mi Lin

**Affiliations:** 1 College of Physics and Optoelectronic Engineering, Shenzhen University, Shenzhen 518060, China; 2 THz Technical Research Center of Shenzhen University, Shenzhen University, Shenzhen 518060, China

**Keywords:** surface plasmon polaritons, fan-shaped cavity, tunable resonances, coupled-cavity system, mode interference, finite element method

## Abstract

Multiple resonance modes have important applications since they can provide multi-frequency operation for devices and bring great flexibility in practice. In this paper, based on a fan-shaped cavity coupled to a metal-isolator-metal (MIM) waveguide, a new kind of ultracompact plasmonic nanostructure is proposed to realize multiple resonance modes with dense distribution in a broad spectral range, and demonstrated through finite-element method (FEM) simulations. As many as ten resonance modes with an average interval of about 30 nm are obtained. They originate from the coexistence and interference of three types of basic modes in the fan-shaped cavity, i.e., the ring-waveguide modes, the modes in a ring array of periodic air grooves, and the metal-core-cavity modes. The dependence of resonance modes on structure parameters is investigated, which can provide an effective guide for choosing appropriate multiple-resonance-mode structures. Furthermore, by means of adjusting the geometrical asymmetry induced by the axial offset of the metal core in the fan-shaped cavity, the resonance modes can be effectively modulated, and some new modes appear because the wave path in the cavity is changed. The result proposes a novel way to create multiple resonance modes in plasmonic nanostructures, providing additional degrees of freedom for tailoring the resonance spectra and promising applications in various plasmonic devices, such as optical filters, ultrafast switches, biochemical sensors, and data storages.

## 1. Introduction

Surface plasmon polaritons (SPPs) are electromagnetic waves propagating along metal-dielectric interfaces and decaying drastically along the vertical direction of metal surfaces, generated through the interaction between incident light and free electrons in metal [1,2,3,4,5]. Much attention has been devoted to SPP structures since they have many attractive capabilities, such as overcoming the optical diffraction limit, forming strongly localized fields on metal-dielectric surfaces, and manipulating light at nanoscale [6,7,8,9,10,11,12,13]. Due to these unique characteristics, a variety of SPP devices with special functions have been theoretically proposed and experimentally demonstrated, such as optical modulators, logic processors, power splitters, optical filters, and biochemical sensors [14,15,16,17,18,19,20,21]. Interestingly, SPP is regarded as one of the most promising technologies for realizing all-optical on-chip integrated systems.

Recently, plasmonic devices with multiple resonance modes have attracted great research interest since they are very useful in applications that need to be operated at different frequencies. As we know, the main method to obtain plasmonic multimodes is to cascade several identical or similar (stretching or shrinking the scale of the size with the configuration unchanged) resonance elements, such as T-typed waveguides, nanodisk cavities, or rectangular resonators [22,23,24,25,26,27,28,29]. The disadvantage of this method is that identical or similar resonance elements generally have common optical properties, which may lead to monotonous coupling in structure. In addition, if one would want to enhance the number of modes, more resonance elements are generally required. This would increase the size of the structure and bring inconvenience to the integration of devices. 

In this paper, in order to solve the problem, one strategy is to use completely different types of resonance elements to realize multiple SPP resonance modes. There are two points that need to be considered. Firstly, for a single type of basic element, it can only support a few resonance modes in a certain range of wavelength. However, their optical properties are quite different, which is an advantage that can be applied. If these basic elements are effectively assembled as a whole, it is possible to offer great freedom to obtain rich coupling patterns, which may greatly enhance the number of resonance modes. Secondly, when different elements are properly selected and matched, devices can be very compact due to the complementarity in structure of these elements.

Based on the above proposal, three kinds of different basic elements, i.e., a ring waveguide, a ring array of periodic metal-air grooves, and a metal-core cavity, are selected to construct a compact fan-shaped cavity, and due to the integration effect and the interference among them, as many as ten resonance modes with dense distributions in a broad spectral range are obtained. The influence of structure parameters on resonance modes is analyzed in detail. Furthermore, the resonance modes are effectively modulated through changing the geometrical asymmetry in the fan-shaped cavity.

## 2. Structure Design

**Figure 1:**
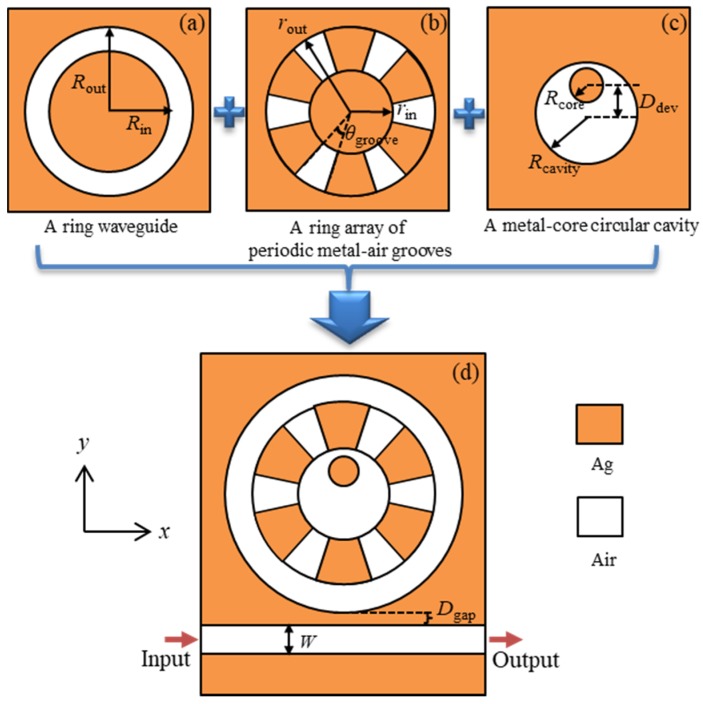
The cross-section schematic diagrams of plasmonic nanostructures; (**a**) a ring waveguide, (**b**) a ring array of periodic metal-air grooves, (**c**) a metal-core circular cavity, and (**d**) a fan-shaped cavity formed by the three basic elements above and a side-coupled straight waveguide.

As we know, localized surface plasmons sustained by a metal-isolator-metal (MIM) nanostructure strongly depend on the geometry and size of the structure. By properly selecting and designing the structure, it is possible to realize multiple resonance modes in a compact structure. As shown in Figure 1, we can see a cross-section schematic diagram of the two-dimensional plasmonic nanostructure proposed. The orange and white denote silver and air, respectively.

Three kinds of different elements were selected, i.e., the ring waveguide in Figure 1a, the ring array of periodic metal-air grooves in Figure 1b, and the metal-core circular cavity in Figure 1c, to form a fan-shaped cavity. In order to input and output electromagnetic waves, a MIM straight waveguide was side-coupled to the fan-shaped cavity with the width *W* = 70 nm to ensure that only the fundamental transverse magnetic (TM) mode was supported. The vertical gap between the fan-shaped cavity and waveguide was set as *D*_gap_ = 20 nm. The inner and outer radii of the ring waveguide were set as *R*_in_ = 400 nm and *R*_out_ = 535 nm, respectively. For convenience, the width of the ring waveguide was defined as *W*_ring_ = *R*_out_ − *R*_in_. For the ring array of periodic metal-air grooves, all the inner edges of the grooves were distributed on a circle with a radius *r*_in_ = 180 nm and the outer edges were on the other circle with a radius *r*_out_ = 400 nm. Thus, the height of grooves was defined as *H*_groove_ = *r*_out_ − *r*_in_. The center angle of the air grooves was *θ*_groove_ = 25°. The radius of the metal-core circular cavity was denoted as *R*_cavity_. It had *R*_cavity_ = *r*_in_. The metal core had a radius *R*_core_ = 85 nm. Furthermore, in order to adjust the resonance modes, geometrical asymmetry was introduced in the fan-shaped cavity by moving the metal core away from the center of the cavity with a parameter *D*_dev_ denoting the axial offset between the two axes of the metal core and the circular cavity.

For the silver material, the frequency-dependent complex relative permittivity *ε*_Ag_(*ω*) was characterized by the Drude mode [30,31]:

(1) $$\varepsilon_{\mathrm{Ag}}(\omega )=\varepsilon_{\infty }-\frac{\omega_{p}^{2}}{\omega (\omega +i\gamma )}$$

where *ε*_∞_ is the dielectric constant at infinite frequency, *γ* is the electron collision frequency, *ω* is the frequency of incident light, and *ω_p_* is the bulk plasma frequency. The values of the parameters were *ε*_∞_ = 3.7, *ω*_p_ = 1.38 × 10^16^ Hz, and *γ* = 2.73 × 10^13^ Hz. Note that, the Drude model provides an effective description of the free carrier response in metal when the band structure effects and the underlying electron interactions are neglected. For the wavelengths ranged from about 500 nm to 1500 nm in our structure, the Drude model acted as a simple and good approximation for Ag. Beyond the above range, the extended Drude model was more suitable [32]. It is worthwhile to mention that in reality, the experimental measurements give quite disperse values for the permittivity, such as the experimental data in the reference [32]. For other noble metals, gold can also be chosen [33].

The transmission properties of the fan-shaped structure were investigated using Comsol Multiphysics software (5.3a, Stockholm, Sweden) based on finite-element method (FEM). A transverse magnetic continuous wave was excited at the left side of the straight waveguide. The transmission was detected at the right side of the straight waveguide and determined as *P*_out_/*P*_in_, where *P*_in_ and *P*_out_ stand for the input and output energy flows obtained by integrating the *x* component of the Poynting vector over the cross section of the left and right waveguide ports. The structure surrounded by perfectly matched layers was divided into about 6.5 × 10^4^ cell grids to ensure the accuracy of calculation.

## 3. Results and Discussions

### 3.1. The Coupling Mechanism of the Multiple Resonance Modes

**Figure 2:**
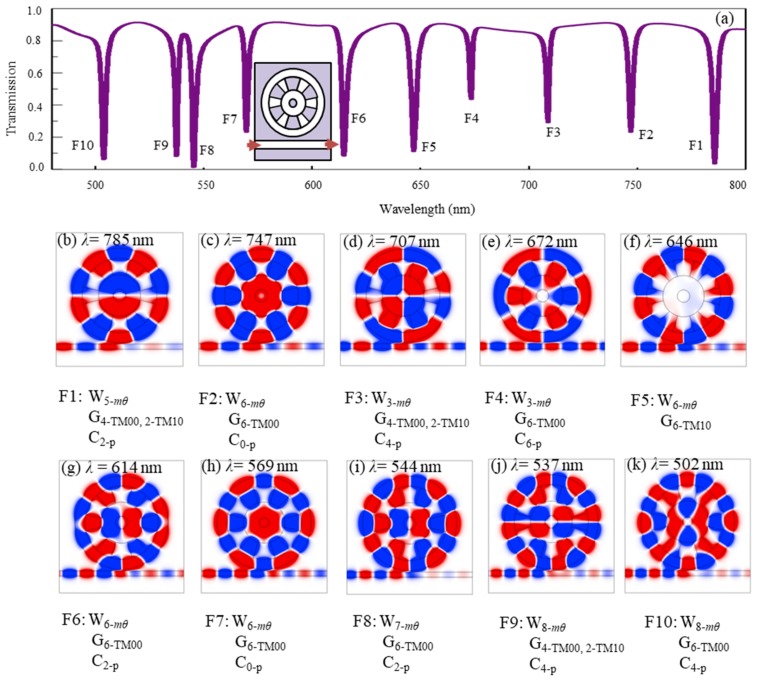
(**a**) The transmission of the fan-shaped structure with *D*_dev_ = 0, and the *H_z_*-field distributions at the transmission dips of (**b**) F1 with *λ* = 785 nm, (**c**) F2 with *λ* = 747 nm, (**d**) F3 with *λ* = 707 nm, (**e**) F4 with *λ* = 672 nm, (**f**) F5 with *λ* = 646 nm, (**g**) F6 with *λ* = 614 nm, (**h**) F7 with *λ* = 569 nm, (**i**) F8 with *λ* = 544 nm, (**j**) F9 with *λ* = 537 nm, (**k**) F10 with *λ* = 502 nm.

Figure 2a shows the transmission spectrum of the fan-shaped cavity coupled with a straight waveguide. The symmetric structure was considered with *D_dev_* = 0, whose detailed structure is illustrated by the inset in Figure 2a. We can see that as many as ten narrow transmission dips appeared at the wavelengths *λ*_F1_ = 785 nm, *λ*_F2_ = 747 nm, *λ*_F3_ = 707 nm, *λ*_F4_ = 672 nm, *λ*_F5_ = 646 nm, *λ*_F6_ = 614 nm, *λ*_F7_ = 569 nm, *λ*_F8_ = 544 nm, *λ*_F9_ = 537 nm and *λ*_F10_ = 502 nm in a broad high-transmission band, in which F1–F10 denotes the ten resonance modes, respectively. They exhibit a very dense distribution with an average interval of about 30 nm in the range of *λ* = 502 nm to *λ* = 785 nm. In order to investigate the property of the resonance modes, the quality factor of Q = 2πνE/P = ν/Δν was considered [34]. Here, E is the stored energy in the cavity, and P is the power dissipated. ν = c/λ is the resonance frequency of the cavity, λ is the resonance wavelength, and c is the speed of light. ∆ν is the 3-dB bandwidth of a resonance dip of the cavity. The quality factors of the ten resonance modes were calculated as Q_F1_ = 320, Q_F2_ = 294, Q_F3_ = 442, Q_F4_ = 390, Q_F5_ = 232, Q_F6_ = 252, Q_F7_ = 321, Q_F8_ = 299, Q_F9_ = 375, Q_F10_ = 252, respectively. The high-quality factors show that the resonance modes have very narrow line-shapes.

In order to determine the origin of the multiple resonance modes, the *H*_z_-field distributions at the resonance wavelengths are shown in Figure 2b–k. As expected, complex standing waves with different field patterns were formed in the fan-shaped cavity. The result shows that the fan-shaped cavity indeed can be divided into three parts, including the ring waveguide in the outer part, the ring array of periodic metal-air grooves in the middle part, and the metal-core circular cavity in the inner part.

To further understand the mechanism of the coupling modes, the transmission characteristics of the three basic elements are to be investigated separately.

**Figure 3:**
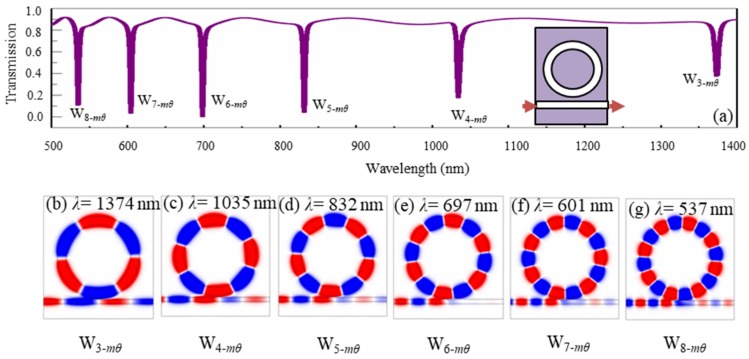
(**a**) The transmission of the ring waveguide coupled with a straight waveguide, and the *H_z_*-field distributions at the transmission dips of (**b**) W_3-_*_m__θ_* with *λ* = 1374 nm, (**c**) W_4-_*_m__θ_* with *λ* = 1035 nm, (**d**) W_5-_*_m__θ_* with *λ* = 832 nm, (**e**) W_6-_*_m__θ_* with *λ* = 697 nm, (**f**) W_7-_*_m__θ_* with *λ* = 601 nm, and (**g**) W_8-_*_m__θ_* with *λ* = 537 nm.

Firstly, Figure 3a shows the transmission spectrum of a ring waveguide coupled with a straight waveguide. The detailed structure is illustrated by the inset in Figure 3a. The inner and outer radii of the ring waveguide were 400 nm and 535 nm, respectively. The vertical gap between the ring and straight waveguides was set as 20 nm. We can see that six narrow transmission dips were formed at the wavelengths *λ* = 1374 nm, *λ* = 1035 nm, *λ* = 832 nm, *λ* = 697 nm, *λ* = 601 nm, and *λ* = 537 nm. The *H*_z_-field distributions at the resonance wavelengths are shown in Figure 3b–g. Due to the resonance effect in the ring waveguide, we can see that the six resonance modes had an azimuthal quantum number *m_θ_* of 6/2, 8/2, 10/2, 12/2, 14/2 and 16/2, respectively, which means the waves travelled by 3, 4, 5, 6, 7, and 8 periods azimuthally in the ring waveguides, respectively, for each round. It is evident that a long resonance wavelength corresponded to a lower azimuthal quantum number of the resonance wave, which satisfied the principle of resonance cavity. Theoretically, we should have (*m_θ_* λ) being equal to the circumference of the ring cavity multiplied by the effective index for each resonance wavelength λ. However, due to dispersion of the metal, the effective circumference was related to the wavelength and thus the ratio of quantum numbers for different modes had a small difference to the ratio of wavelengths for the modes. For example, the ratio of quantum numbers in Figure 3b,e is two, which has a small difference with the ratio 1.971 of the wavelengths in Figure 3b,e. For convenience, the six resonance modes are denoted as W_3-_*_m__θ_*, W_4-_*_m__θ_*, W_5-_*_m__θ_*, W_6-_*_m__θ_*, W_7-_*_m__θ_*, W_8-_*_m__θ_*, respectively.

**Figure 4:**
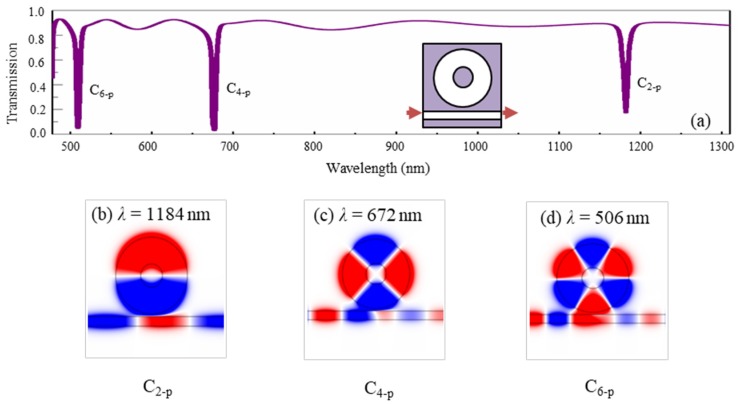
(**a**) The transmission spectrum of the metal-core circular cavity coupled with a straight waveguide, and the *H_z_*-field distributions at the transmission dips of (**b**) C_2-p_ with *λ* = 1184 nm, (**c**) C_4-p_ with *λ* = 672 nm, and (**d**) C_6-p_ with *λ* = 506 nm.

Secondly, Figure 4a shows the transmission spectrum of the metal-core circular cavity coupled with a straight waveguide. The detailed structure is given in the inset. The radius of the metal-core circular cavity was 180 nm and the metal core had a radius of 85 nm. The vertical gap between the circular cavity and straight waveguides was 20 nm. It can be found that three narrow transmission dips appeared at the wavelengths *λ* = 1184 nm, *λ* = 672 nm, and *λ* = 506 nm. The *H*_z_-field distributions at the resonance wavelengths are shown in Figure 4b–d. Obviously, the three resonance modes corresponded to dipole, quadrupole, and hexapole modes, which can be denoted by C_2-p_, C_4-p_ and C_6-p_, respectively.

**Figure 5:**
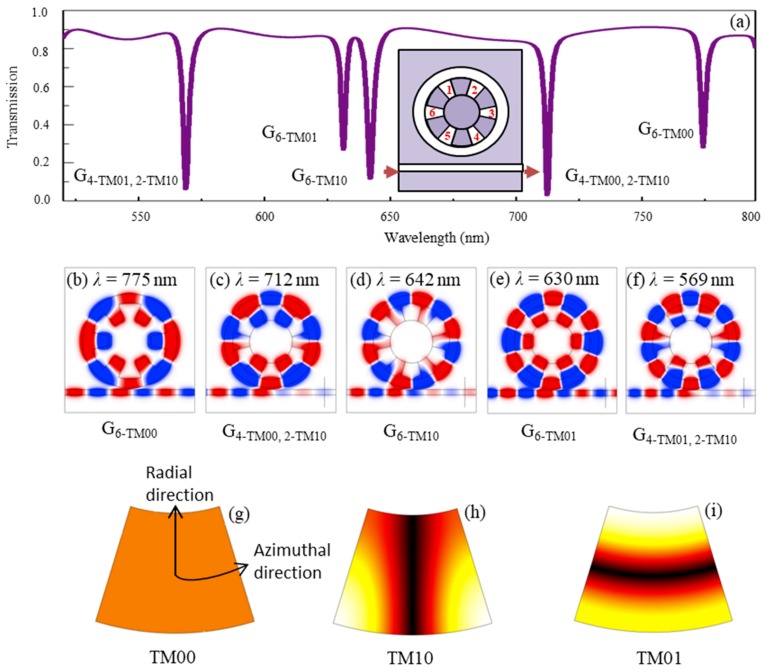
(**a**) The transmission spectrum of the periodic metal-air grooves surrounded by a ring waveguide and coupled with a straight waveguide, the *H_z_*-field distributions at the transmission dips of (**b**) G_6-TM00_ with *λ* = 775 nm, (**c**) G_4-TM00,2-TM10_ with *λ* = 712 nm, (**d**) G_6-TM10_ with *λ* = 642 nm, (**e**) G_6-TM01_ with *λ* = 630 nm, and (**f**) G_4-TM01,2-TM10_ with *λ* = 569 nm, and the three fundamental eigenmodes existed in the single air groove in metal for (**g**) TM_00_ mode, (**h**) TM_10_ mode, and (**i**) TM_01_ mode.

Thirdly, Figure 5a shows the transmission spectrum of the ring array of periodic metal-air grooves surrounded by a ring waveguide and coupled with a straight waveguide. The detailed structure is illustrated in the inset. For convenience, the six periodic air grooves are marked as 1–6. The inner and outer radii of the ring waveguide were 400 nm and 535 nm, respectively. The height and center angle of the air grooves was 220 nm and 25°, respectively. The vertical gap between the ring and straight waveguides was 20 nm. It should be emphasized that the ring waveguide was used in the structure in order to excite the resonance modes in the air grooves. The result showed that five narrow transmission dips were formed at wavelengths *λ* = 775 nm, *λ* = 712 nm, *λ* = 642 nm, *λ* = 630 nm, and *λ* = 569 nm.

Furthermore, the *H*_z_-field distributions at the resonance wavelengths are shown in Figure 5b–f. It can be shown that different field patterns were formed in the ring array of periodic air grooves. In order to explain the above optical property, it is necessary to investigate the single air groove in metal. As shown in Figure 5g–i, three fundamental eigenmodes existed in the single air groove. According to the field patterns, the modes can be expressed as TM*_mn_*, where *m* and *n* denote the order numbers of the modes in the azimuthal and radial directions, respectively. Thus, the three eigenmodes can be also denoted as TM_00_, TM_10_ and TM_01_, respectively, which means that TM_00_ in Figure 5g is a zero-pole mode, TM_10_ in Figure 5h is the 1st-order resonance mode along the azimuthal direction, and TM_01_ in Figure 5i is the 1st-order resonance mode along the radial direction. Based on the above analysis, the result in Figure 5b–f indicated that, different field patterns were excited in the ring array of periodic air grooves, whose details are given in the following. There are six TM_00_ modes (air grooves 1–6) for *λ* = 775 nm, four TM_00_ modes (air grooves 1, 2, 4 and 5) and two TM_10_ modes (air grooves 3 and 6) for *λ* = 712 nm, six TM_10_ modes (air grooves 1–6) for *λ* = 642 nm, six TM_01_ modes (air grooves 1–6) for *λ* = 630 nm, four TM_01_ modes (air grooves 1, 2, 4 and 5) and two TM_10_ modes (air grooves 3 and 6) for *λ* = 569 nm. For simplicity, the five resonance dips are denoted as G_6-TM00_, G_4-TM00,2-TM10_, G_6-TM10_, G_6-TM01_, and G_4-TM01,2-TM10_, respectively.

By comparing Figure 2, Figure 3, Figure 4, Figure 5, it revealed that the resonance modes F1–F10 with extremely narrow dips in Figure 2 resulted from the interference of the three kinds of different modes from the ring waveguide in Figure 3, the metal-core circular cavity in Figure 4, and the ring array of periodic air grooves in Figure 5. Details are given as follows. The modes F1–F10 originate from the coupling of (W_5-_*_m__θ_*, G_4-TM00,2-TM10_, C_2-p_), (W_6-_*_m__θ_*, G_6-TM00_, C_0-p_), (W_3-_*_m__θ_*, G_4-TM00,2-TM10_, C_4-p_), (W_3-_*_m__θ_*, G_6-TM__00_, C_6-p_), (W_6-_*_m__θ_*, G_6-TM10_), (W_6-_*_m__θ_*, G_6-TM00_, C_2-p_), (W_6-_*_m__θ_*, G_6-TM00_, C_0-p_), (W_7-_*_m__θ_*, G_6-TM00_, C_2-p_), (W_8-_*_m__θ_*, G_4-TM00,2-TM10_, C_4-p_) and (W_8-_*_m__θ_*, G_6-TM00_, C_4-p_), respectively. We found that the single ring waveguide, the metal-core circular cavity, and the ring array of periodic air grooves could only support fewer resonance modes in a certain range of wavelength. However, when they are effectively assembled as a whole, not only could abundant coupling patterns be obtained, but also the device was quite compact due to their complementarity in structure.

The fan-shaped structure has promising applications in various plasmonic devices. For example, it can be used as optical filter or ultrafast switch due to the sharp line-shapes. Also, it can be applied in biochemical detecting or sensing since the transmission is highly sensitive to the change of the background material.

### 3.2. The Dependence of the Resonance Modes on Structure Parameters

In the following, we investigated the influence of structure parameters on the resonance modes. The investigated parameters included (1) the width *W*_ring_ of the ring waveguide, (2) the radius *R*_cavity_ of the metal-core circular cavity, and (3) the height *H*_groove_ of the periodic air grooves. Such parameters were important to determine the geometry of the fan-shaped cavity.

**Figure 6:**
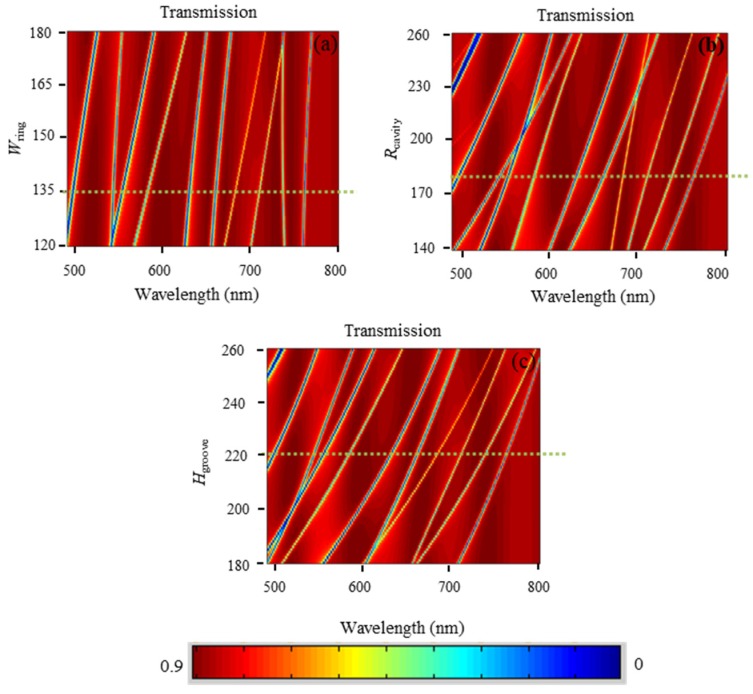
The wavelength shifts of the transmission dips when (**a**) the width *W*_ring_ of the ring waveguide changed from 120 nm to 180 nm, (**b**) the radius *R*_cavity_ of the metal-core circular cavity changed from 140 nm to 260 nm, and (**c**) the height *H*_groove_ of the periodic air grooves changed from 180 nm to 260 nm.

Figure 6a shows the optical response of multiple resonance modes of the fan-shaped structure when the width *W*_ring_ of the ring waveguide changed. We can find that the transmission dips had obvious red shifts when *W*_ring_ increased from 120 nm to 180 nm with the inner radius of the ring waveguide unchanged (*R*_in_ = 400 nm). On the other hand, the red shift also occurred when the radius *R*_cavity_ of the metal-core circular cavity changed from 140 nm to 260 nm, as shown in Figure 6b. These phenomena can be explained as follows. Both the ring waveguide and the metal-core circular cavity can be regarded as ring resonators that exhibited band-stop characteristics. The resonance wavelength *λ*_p_ was determined by the following formula for a Fabry–Perot cavity [34],

(2) $$\lambda_{p}=\frac{n_{s}L_{0}}{m-\frac{\varphi }{2\pi }},\begin{array}{cc} & m=1,2,3\cdots \end{array}$$

where *n*_s_ is the real part of the effect refractive index of the ring resonator, *m* is the resonance order, *L*_0_ is the perimeter of cavity, and *φ* is the phase change due to the reflection of cavity. For the ring cavity discussed in this paper, the above Equation (2) can still be applied, but *φ* becomes the extra phase change when the wave goes over the coupling region between the ring cavity and the horizontal waveguide. It can be found from Equation (2) that *λ*_p_ is proportional to *L*_0_ when the parameters *n*_s_, *m* and *φ* are fixed. If *W*_ring_ or *R*_cavity_ becomes larger, it is equivalent to an increase in the perimeter of cavity, which leads to a larger resonance wavelength. Therefore, the red shifts are formed. 

It should be noted that the effective index *n_s_* was related to the mode pattern in the cavity, i.e., the cavity had a different effective index for different modes. Therefore, the resonance modes in the fan-structure have great dependence on the structure parameters.

Figure 6c shows the optical response of the multiple resonance modes of the fan-shaped structure when the height *H*_groove_ of the periodic air grooves changed. It shows that when *H*_groove_ increased from 180 nm to 260 nm with the angle of the periodic air grooves fixed at *θ*_groove_ = 25°, the transmission dips also had obvious red shifts. This was because when *H*_groove_ was increased, it meant that the space of air grooves was enlarged, which resulted in the movements of resonance modes to larger wavelengths.

It can be concluded from the above results that the resonance modes in the fan-structure have great dependence on structure parameters. There are three main reasons for this. The first is that the size of the structure is in a comparable order of magnitude to the working wavelength. The second is that, there are resonances in the structure, so that the transmission is very sensitive to changes in structure. The third is that resonance modes resulted from the integration and interference of the three basic elements, i.e., the ring waveguide, the metal-core circular cavity, and the ring array of periodic air grooves. Therefore, the structure parameters have an important influence on the resonance modes.

By analyzing Figure 6, in order to obtain multiple resonance modes with proper mode intervals, the parameters *W*_ring_ = 135 nm_,_ *R*_cavity_ = 180 nm_,_ *H*_groove_ = 220 nm can be selected as an example, as shown by the yellow dotted lines in Figure 6, leading to a correspondent result shown in Figure 2. For different applications, other parameters can be selected flexibly according to the characteristics of transmission in Figure 6. These results can provide effective guidance for choosing appropriate multiple-resonance-mode structures.

### 3.3. The Tunability of Resonance Modes by Adjusting the Geometrical Asymmetry

Following this, we studied the tunability of the plasmonic resonance modes in the fan-shaped structure. The electromagnetic modes in the straight waveguide, the ring waveguide, the ring array of periodic air grooves, and the metal-core cavity can be regarded as states 0〉, 1〉, 2〉 and 3〉, respectively. As a result, the coupling process is expressed as 0〉⟶1〉⟶2〉⟶3〉⟶2〉⟶1〉⟶0〉. When adjusting the axis offset *D*_dev_ of the metal core, the symmetry was destroyed in the structure. The increase of the geometric asymmetry corresponded to a further increase of the interaction between the waves in the inner and outer parts of the metal-core circular cavity, thereby the field distribution was greatly affected, leading to the change in the coupling between 2〉 and 3〉. Then the coupling between 1〉 and 2〉; and between 0〉 and 1〉 were also influenced. Therefore, the transmission properties can be tuned easily.

**Figure 7:**
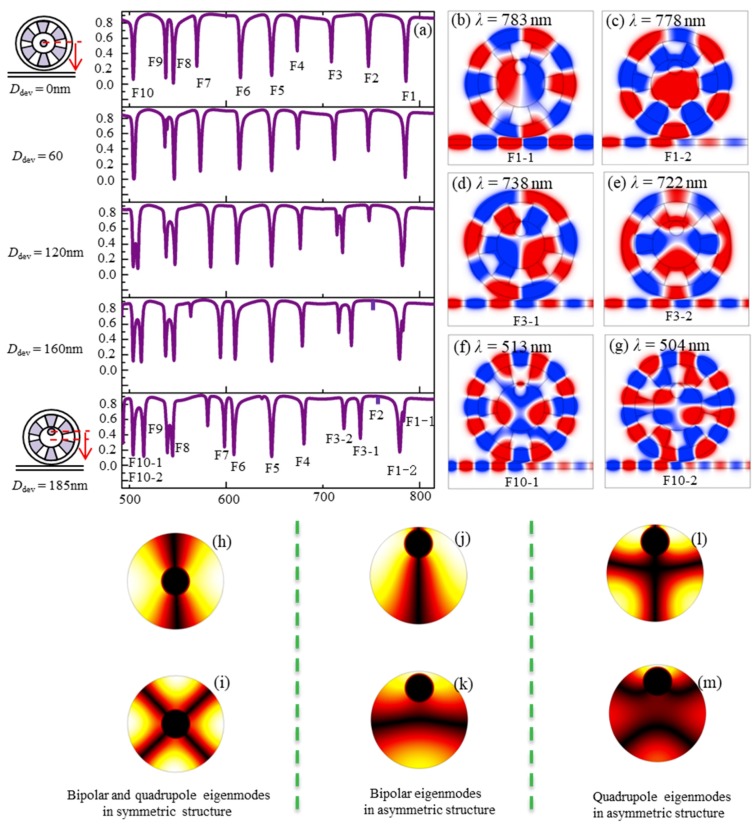
(**a**) The influence of the offset distance *D*_dev_ of the metal core on the transmission spectra. The simulated magnetic-field *H_z_* distributions at the transmission dips of modes (**b**) F1-1 with *λ* = 783 nm, (**c**) F1-2 with *λ* = 778 nm, (**d**) F3-1 with *λ* = 738 nm, (**e**) F3-2 with *λ* = 722 nm, (**f**) F10-1 with *λ* = 513 nm, and (**g**) F10-2 with *λ* = 504 nm. The |*H*| distributions of the metal-core cavity for (**h**) bipole and (**i**) quadrupole eigenmodes in the symmetric structure with *D*_dev_ = 0, and in the asymmetric structure with *D*_dev_ = 185 nm, (**j**,**k**) bipole eigenmodes, and (**l**,**m**) quadrupole eigenmodes.

In order to understand how the geometrical asymmetry affected the resonance modes in detail, a plot of the transmission versus the offset distance *D*_dev_ and the wavelength is shown in Figure 7a. When *D*_dev_ increased from 0 nm to 185 nm, the result revealed that all the resonance modes except F5 had obvious changes with the increase of *D*_dev_. The modes F2, F4 and F6–F9 exhibited wavelength shifts with displacements of 13 nm, 6 nm, −8 nm, 30 nm, −3 nm and 4 nm, respectively. Especially, when *D*_dev_ = 185 nm, some new resonance modes were generated. The modes F1, F3 and F10 split into the modes (F1-1, F1-2), (F3-1, F3-2) and (F10-1, F10-2), respectively, whose *H*_z_-field distributions are shown in Figure 7b–g. This is because once the symmetry of the structure was destroyed, new eigenmodes appeared in the metal-core cavity, which can be found by comparing the bipolar and quadrupole eigenmodes in the two cases of symmetric (*D*_dev_ = 0 nm) and asymmetric (*D*_dev_ = 185 nm) structures shown in Figure 7h–m. In addition, the F5 mode was weakly affected by the change of *D*_dev_ since the field distributions in the metal-core circular cavity and the periodic air grooves were very weak at *λ* = 646 nm (mode F5), as can be seen from Figure 2f.

## 4. Conclusions

In conclusion, we have demonstrated the existence of multiple plasmonic resonance modes in a new kind of ultracompact fan-shaped nanostructure. As many as ten resonance modes with an average interval of about 30 nm were generated in a broad optical spectral range, originating from the interference of three types of different basic modes from the fan-shaped structure, i.e., ring-waveguide modes, modes in the ring array of periodic-air-grooves, and metal-core-cavity modes. The dependence of resonance modes on structure parameters was investigated, which can provide a reference for choosing the appropriate multiple-resonance-mode structures. Furthermore, adjusting the geometrical asymmetry can effectively modulate the resonance modes, and some new modes appeared, which can provide additional degree of freedom for tailoring the resonance spectra. The proposed plasmonic structure has vital applications in optical filters, ultrafast switches, biochemical nanosensors, data storages, and so on. In addition, if there were defects or structural randomness in the system, it would greatly influence the resonance wavelengths. The influence was closely related to the types, sizes, numbers, and locations of the defects, and the degree of randomness. This was highly meaningful for practical applications, and it needs to be studied separately and systematically.
